# A non-invasive ultrasound imaging method to measure acute radiation-induced bladder wall thickening in rats

**DOI:** 10.1186/s13014-020-01684-3

**Published:** 2020-10-17

**Authors:** Antonello E. Spinelli, Andrea Bresolin, Stefania Zuppone, Laura Perani, Giuseppe Fallara, Nadia Di Muzio, Riccardo Vago, Claudio Fiorino, Cesare Cozzarini

**Affiliations:** 1 Experimental Imaging Centre, IRCCS San Raffaele Scientific Institute, Via Olgettina, 60, 20132 Milan, Italy; 2 Medical Physics, IRCCS San Raffaele Scientific Institute, Milan, Italy; 3 Fondazione Centro San Raffaele, Milan, Italy; 4 Urological Research Institute, IRCCS San Raffaele Scientific Institute, Milan, Italy; 5 Radiation Oncology, IRCCS San Raffaele Scientific Institute, Milan, Italy; 6 University Vita-Salute San Raffaele, Milan, Italy

**Keywords:** Animal model, Radiation cystitis, Bladder wall thickness, Radiotherapy, Ultrasound imaging

## Abstract

**Background:**

Methods for the non-invasive quantification of changes in bladder wall thickness as potential predictors of radiation cystitis in pre-clinical research would be desirable. The use of ultrasound for this aim seems promising, but is still relatively unexplored. A method using ultrasound for bladder wall thickness quantification in rats was developed and applied to measure early radiation-induced bladder wall thickness changes.

**Methods:**

Two groups (n = 9 each) of female Fischer rats were treated with a single radiation dose of 25–30 and 35–40 Gy respectively, using an image-guided micro-irradiator; six untreated rats were monitored as a control group. Empty, half-filled and fully-filled bladder volumes were determined for four non-irradiated rats by measuring axes from ultrasound 3D-images and applying the ellipsoid formula. Mean bladder wall thickness was estimated for both ventral and dorsal bladder sides through the measurement of the bladder wall area along a segment of 4 mm in the central sagittal scan, in order to minimize operator-dependence on the measurement position. Ultrasound acquisitions of all fully-filled rat bladders were also acquired immediately before, and 4 and 28 days after irradiation. Mean bladder wall thickness normalized to the baseline value and corrected for filling were then used to evaluate acute bladder wall thickening and to quantify the dose–effect.

**Results:**

The relationship between mean bladder wall thickness and volume in unirradiated rats showed that for a bladder volume > 1.5 mL the bladder wall thickness is almost constant and equal to 0.30 mm with variations within ± 15%. The average ratios between post and pre irradiation showed a dose–effect relationship. Bladder wall thickening was observed for the 25–30 Gy and 35–40 Gy groups in 2/9 (22%) and 5/9 (56%) cases at day 4 and in 4/9 (44%) and 8/9 (89%) cases at day 28, respectively. The two groups showed significantly different bladder wall thickness both relative to the control group (*p* < 0.0001) and between them (*p* = 0.022). The bladder wall thickness increment was on average 1.32 ± 0.41, and was 1.30 ± 0.21 after 25–30 Gy and 1.47 ± 0.29 and 1.90 ± 0.83 after 35–40 Gy at days 4 and 28 respectively.

**Conclusions:**

The feasibility of using ultrasound on a preclinical rat model to detect bladder wall thickness changes after bladder irradiation was demonstrated, and a clear dose–effect relationship was quantified. Although preliminary, these results are promising in addressing the potential role of this non-invasive approach in quantifying radiation cystitis.

## Background

Radiation cystitis (RC) is an inflammatory condition of the bladder [1] induced by radiotherapy (RT) when tumors in the pelvic region are treated, as in the case of prostate cancer. The progression of the radiation-induced bladder damage in humans consists of three main steps: an *acute* phase with recovery usually within a few weeks, a *symptom-free* period whose duration depends on the radiation dose, and, lastly, a chronic, irreversible *late* response, in general arising within a year [2–4]. The modeling of radiation-induced urinary toxicity represents a complex topic [5, 6] which is attracting increasing interest in radiotherapy [7, 8]. A thorough understanding of the mechanisms underlying RC is still largely lacking, and in vivo preclinical research has a decisive role in improving knowledge concerning the causes and, hence, possible solutions to at least limit its clinical impact.

The most used and tested tool for the evaluation of in vivo radiation-induced urinary functional toxicity in animal models is cystometry [2, 4, 9–11], i.e. the measurement of micturition frequency, pressure and volume. For morphologic evaluations, immunohistochemistry is the gold standard, as it allows both width measurements of bladder layers and the assessment of inflammatory infiltrates, bladder wall fibrosis and urothelial changes [10–15]. Rapid technological advances in recent years have allowed the development of promising new non-invasive methods to evaluate bladder morphology. In this context, the ultrasound imaging system (US) represents an interesting option, as it combines high spatial resolution, non-invasiveness and ease of use. Along with ultrasound we investigated with a preliminary experiment (not included here) the use of 7 T preclinical MRI as an alternative imaging method to measure BWT. However considering the lower spatial resolution that could be achieved with this setup we decided to perform the whole BTW study using ultrasound imaging.

The use of US to measure RC is still unexplored in the preclinical context, although there is a growing interest in the ultrasonography of the urinary bladder for functional and morphologic assessments [16–19], addressing any variation in bladder wall thickness (BWT) as a potential predictor of RC. On the other hand, as in humans [7, 8], BWT may be significantly influenced by variable filling and this issue must be accounted for before potential applications can be assessed.

The aims of this work were, firstly, to establish a reliable protocol to measure bladder wall thickening in rats, and, secondly, to evaluate early BWT changes in rat bladders exposed to increasing levels of radiation doses.

## Methods

### Animals

Twenty-four eight-week-old adult female Fischer rats (CDF, Strain code #002, Charles River Laboratories) were used. The animal protocol was approved by the Italian Institutional Animal Care and Use Committee (IACUC, approval number 698/2015).

In the first phase of the experiment, four untreated rats were used to optimize the US imaging protocol and to investigate the relationship between BWT and bladder filling. In the second phase, two groups composed of nine rats each were irradiated with a single fraction at 25–30 Gy and 35–40 Gy respectively; six additional non-irradiated rats were used as a control group. BWT of each animal was monitored by US scans at different predetermined timings: baseline (defined as one day before irradiation), 4 and 28 days after irradiation.

For both US imaging and irradiation, rats were anesthetized with gaseous anesthesia (2–3% isoflurane and 1 L/min oxygen). Ultrasound acquisitions were performed considering a full bladder condition. Bladder filling was achieved by slowly injecting a saline solution into the organ through a catheter and using a graduated syringe, we injected in the bladder the same amount of physiologic solution. During each ultrasound acquisition the catheter was kept in position in order to prevent bladder voiding. Unlike imaging, the radiation delivery sessions were performed in the empty bladder condition in order to minimize the volume exposed to treatment beams, and to avoid possible adverse events in adjacent organs (i.e*.* bowel and rectum).

### Phase I: Ultrasound acquisitions and measurements

The model was developed by measuring bladder volume and BWT in four non-irradiated rats in empty, half-filled and fully-filled bladder conditions. These measurements were performed using a US imaging system.

The anesthetized rats were previously shaved on the ventral side of the pelvic region with a depilatory cream (Veet, Reckitt Benkiser, Milan, Italy); the rats were then placed in the supine position on a warmed animal stage. The rat bladder was catheterized transurethrally with PE 20 tubing (2 Biological Instruments, Besozzo, VA, Italy) and injected with approximately 1 mL of normal saline. A pre-warmed ultrasound gel (Aquasonic, Parker Laboratories Inc, Fairfield, NJ, USA) was used as a coupling agent between the ultrasound probe and the skin. Two and three dimensional US images in B-mode were performed with Vevo 2100 ultrasonographic system high-frequency linear probe (MicroScan MS 550D; 22–55 MHz; FUJIFILM VisualSonics, Inc., Toronto, ON, Canada). In order to obtain three-dimensional images of the bladder, the scanner was mounted on a Vevo Imaging Station (part of the VisualSonics Vevo Integrated Rail System III) equipped with a 3D motor stage and positioned on the rats’ pelvic region. The 3D images were acquired by computer-controlled translation of the transducer over the entire length of the urinary bladder, acquiring 2D images every 140 μm. The bladder was then scanned in the transverse and sagittal planes. The total time required for US imaging was approximately 15 min/rat.

US images were analyzed off-line using Vevo LAB analysis software (Fujifilm VisualSonics Inc., Toronto, ON, Canada) by a dedicated operator.

#### Bladder filling measurements

**Fig. 1:**
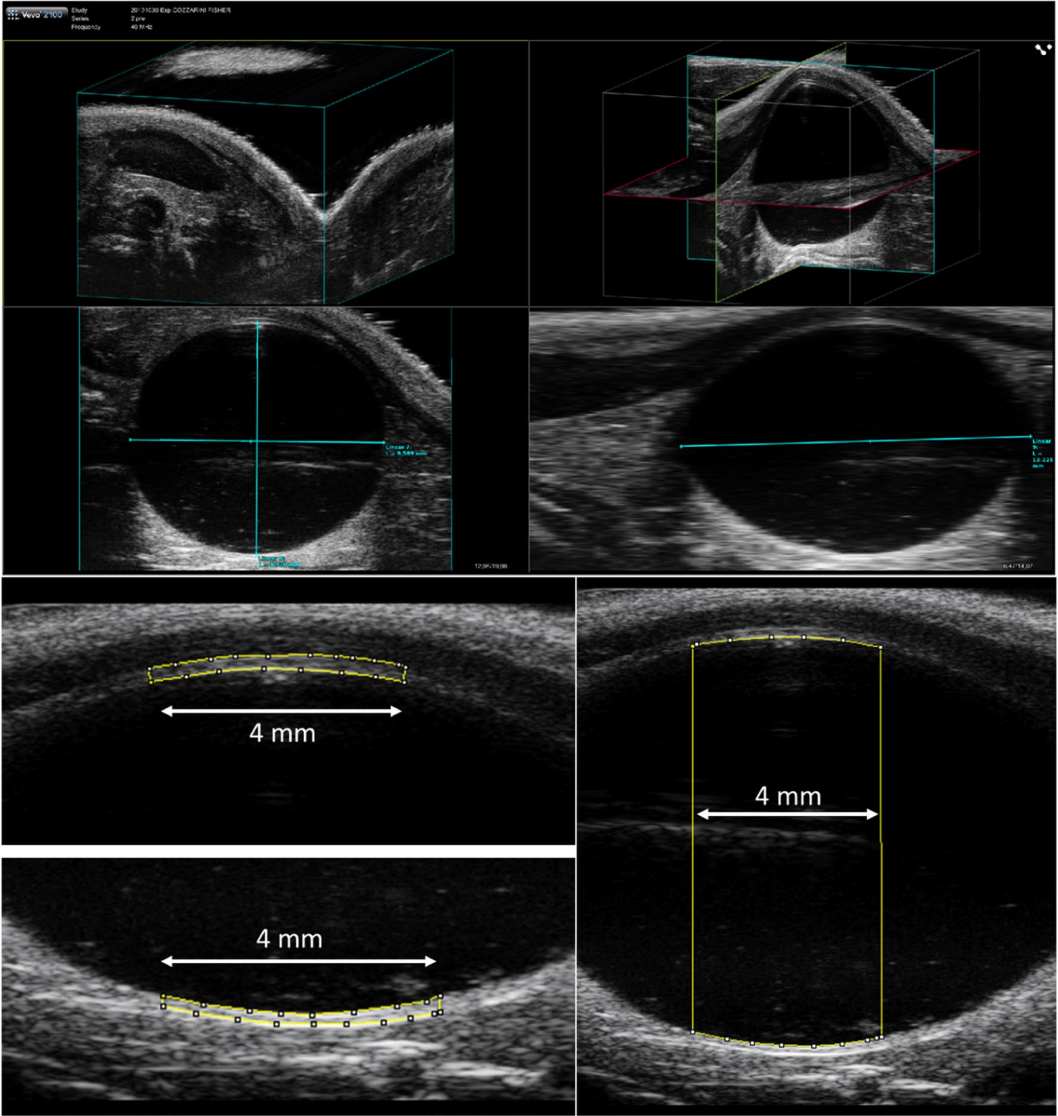
**a** Example of a 3-dimensional ultrasound scan of rat bladder. The light blue segments in the third and fourth quadrants represent the major axes of the ellipsoid. Examples of **b** bladder wall area on the ventral and dorsal side and **c** area inside bladder measured along a segment of 4-mm in the central sagittal ultrasound scan (B-mode)

Bladder filling was estimated through the acquisition of 3D US scans. Bladder volume was determined by measuring the transverse and sagittal bladder axes in the 3D image (Fig. 1a) and applying the following formula in the approximation of an ellipsoid organ:

$$V = \frac{4}{3}\pi abc$$

where $V$ is the ellipsoid volume, and $abc$ the length of its three major axes.

#### Bladder wall thickness measurements

The BWT was estimated through the acquisition in B-mode of sagittal US scans of the maximum bladder section. The mean bladder wall thickness $\left( {BWT_{mean} } \right)$ for both ventral and dorsal sides was defined throughout the measurement of the bladder wall area $\left( {BWA} \right)$ along a segment of 4 mm in the central sagittal scan (as shown in Fig. 1b) according to the relation:

$$BWT_{mean} = \frac{BWA}{{4 mm}}$$

Similarly, the area inside the bladder was measured to assess the mean diameter $\left( {2R_{mean} } \right)$ of the organ as a possible surrogate of the 3D volume estimation (Fig. 1c).

This procedure was repeated for every US image acquisition at each filling condition. The relationship between mean BWT and bladder volume was then plotted and fitted. Bladder image analysis was performed using ImageJ [20].

### Phase II: Irradiation and ultrasound monitoring

In the second phase of the experiment, the model was applied in order to investigate the response of the bladder to different radiation doses. Two groups of nine rats each were irradiated, with six rats used as a control group. The number of animals was suitable to assess a minimum effect size of 1.3 (considering the standard values: $\alpha = 0.05$ and $\beta = 0.20$).

Rats were originally organized in three groups of six animals each to be irradiated at (i) 25 Gy, (ii) 30 Gy and (iii) 35 Gy, respectively. Three additional rats were selected for the group (iv) at 40 Gy, a smaller number given that the radiation effect was expected to be more likely at this higher dose. Unfortunately, one rat in group (i) and two in group (ii) died during the anesthetic procedure since in the setup phase the animals were kept slightly longer under anesthesia. The animals were therefore analyzed in only two groups for the two dose ranges (25–30 Gy and 35–40 Gy) in order to maintain an equal number inside each group and to improve the statistical power of the results.

**Fig. 2:**
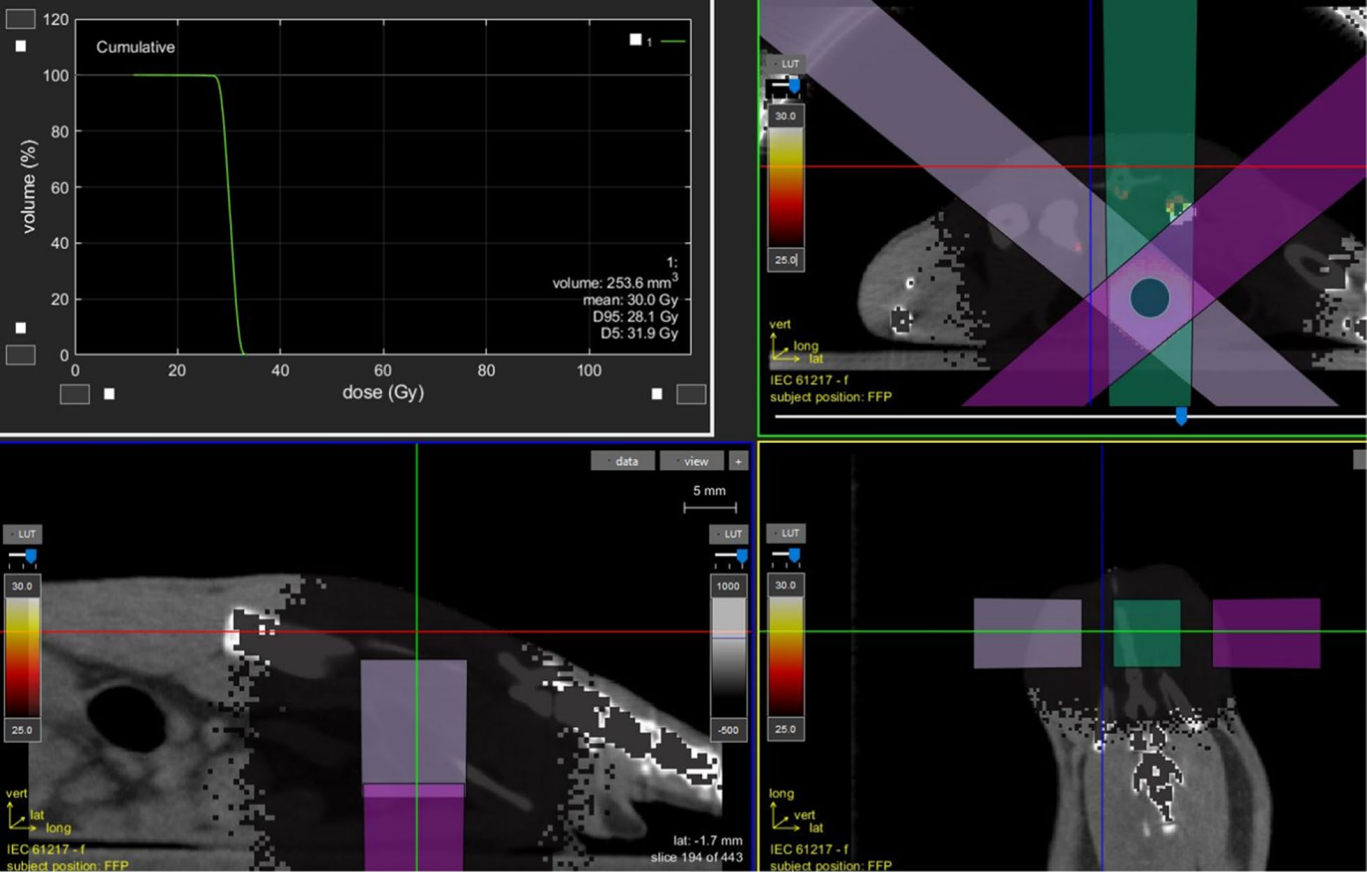
Radiotherapy treatment geometry and, at the top left corner, an example of the dose-volume histogram calculated by a Monte Carlo algorithm for a rat bladder contoured on cone beam CT image

The radiation dose was delivered using a dedicated small animal micro-irradiator (X-RAD225Cx SmART, PXI North Branford, CT, USA) with micro-CBCT guidance. The anesthetized rats were positioned prone on the animal stage and underwent the entire treatment, mimicking the radiotherapy workflow. CBCT images were acquired using the following settings: tube voltage = 40 kVp, current = 5 mA, voxel size = 0.2 mm^3^. The bladder was contoured on the CT scan and three equal-sized dose beams were set at 130°, 180° and 230° angles respectively, as shown in Fig. 2, using a collimator of 10 × 10 mm^2^. Dose distribution was calculated by means of a Monte Carlo algorithm [21] and the mean dose to the bladder was adjusted to the prescribed dose of 25–30 or 35–40 Gy. Irradiation settings were: tube voltage = 225 kVp, current = 13 mA. Delivery time ranged approximately between 2 and 5 min/field and the entire procedure (CT imaging + RT) was performed within 20–25 min/rat.

Bladder wall thickening due to acute radiation effects was investigated with US imaging on days − 1, + 4 and + 28 from RT. The timing of 4 and 28 days was chosen in order to observe a possible biphasic acute response, as described in the literature [2, 3]. Imaging was performed in the full bladder condition in order to maintain measurement set-up reproducibility. $BWT_{mean}$ was measured on the ventral side of bladder. Prior to analysis, the measurements were rescaled as follows:

$$BWT_{ratio}^{{V_{ref} }} = \frac{{BWT_{mean}^{{V_{ref} }} \left( t \right)}}{{BWT_{mean}^{{V_{ref} }} \left( {t_{0} } \right)}} = \frac{{BWT_{mean} \left( t \right) \cdot \frac{2R\left( t \right)}{{2R_{ref} }}}}{{BWT_{mean} \left( {t_{0} } \right) \cdot \frac{{2R\left( {t_{0} } \right)}}{{2R_{ref} }}}} = \frac{{BWT_{mean} \left( t \right) \cdot 2R\left( t \right)}}{{BWT_{mean} \left( {t_{0} } \right) \cdot 2R\left( {t_{0} } \right)}}$$

where $BWT_{ratio}^{{V_{ref} }}$ represents the mean bladder wall thickness normalized to the baseline value at time $t_{0}$ and converted to the corresponding BWT to a reference volume $\left( {V_{ref} } \right)$ and reference radius $\left( {R_{ref} } \right)$. In this way, the amount of change in terms of bladder wall thickness can be considered less biased by inter- and intra-animal variability, as well as by physiological fluctuations due to slightly different bladder fillings. The method for correcting BWT to a proper reference volume has already been proposed and applied by Ke and Kuo for accurate measurement of the human bladder [19]. Since the number of animals in the irradiated groups was designed to be sensitive to an effect size of 1.3, variations in terms of $BWT_{ratio}^{{V_{ref} }}$ above this value were defined as significant BWT changes. Lastly, the Mann–Whitney–Wilcoxon test was performed to assess significant differences between groups.

## Results

### Relationship between bladder wall thickness and bladder filling

**Fig. 3:**
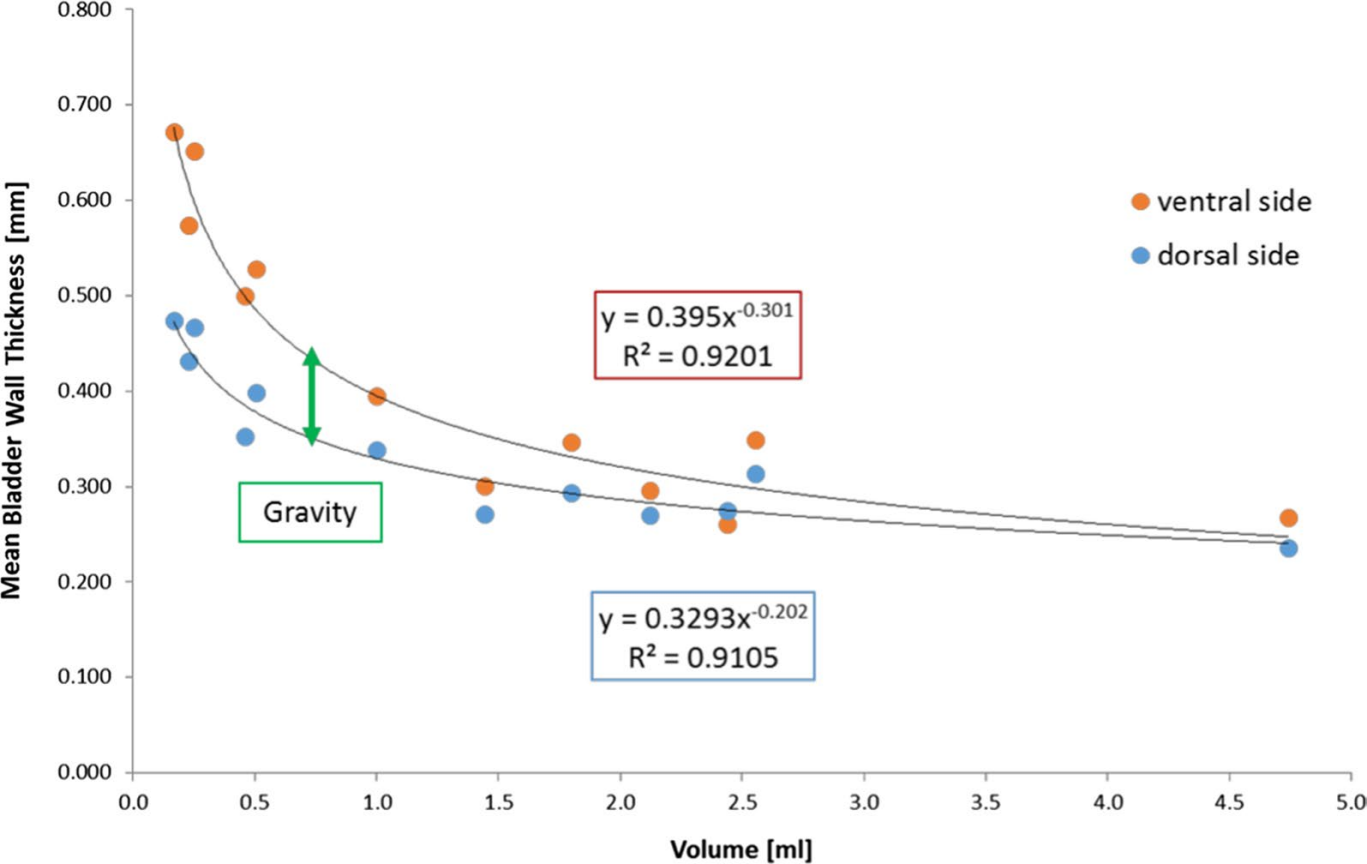
Mean bladder wall thickness of four female non-irradiated Fischer rats against different bladder fillings

Figure 3 shows the relationship between the $BWT_{mean}$ and bladder volume. As expected, a clear trend of a progressive thinning of the bladder wall with increasing bladder fillings was found. In particular, $BWT_{mean}$ on the ventral side was found to be inversely proportional to the cube root of the bladder volume. The quantification of this effect suggests that BWT is almost constant for bladder volume > 1.5 mL: the mean value in the plateau region was 0.30 mm with maximum variations of about 15%. Interestingly, the measurements on the ventral side were nearly always found to be higher than those on the dorsal side, with a decreasing difference as the bladder volume increased, likely due to the difference in gravity effects between the two sides.

### Acute radiation-induced bladder toxicity

**Fig. 4:**
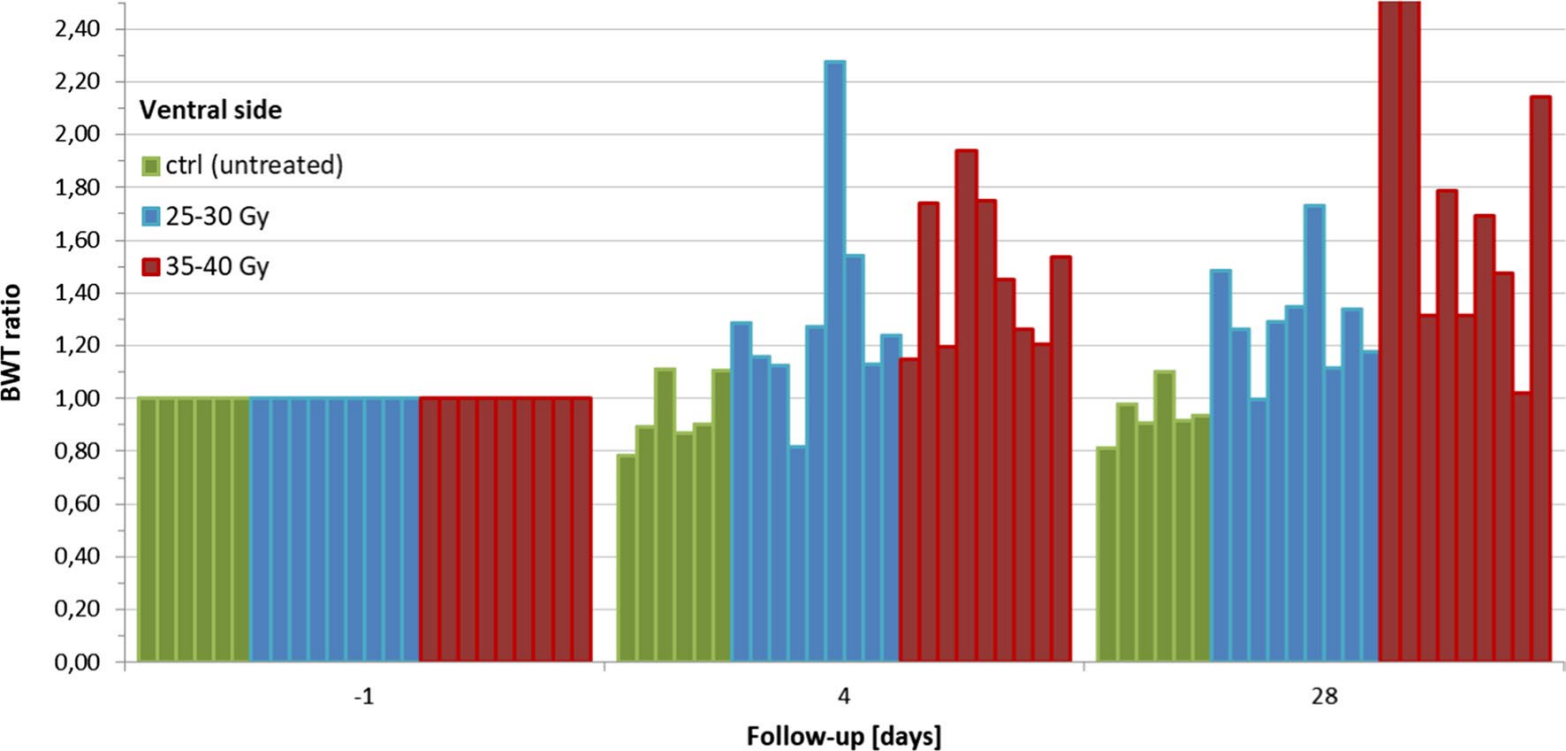
Bladder wall thickness (BWT) normalized to the baseline value based on radiation treatment strength and follow-up timing. The two red bars off the chart correspond to a BWT ratio of 3.60 and 2.78 respectively

Table 1 and Fig. 4 summarize the resulting $BWT_{ratio}^{{V_{ref} }}$ for all animals according to group and the timing of the observations. The variations in the control group ranged between − 22% and + 11% relative to baseline, close to the expected inter-rat variations of BWT with full bladder previously investigated. BWT above the control group maximum value and above the designed effect size was observed for the 25–30 Gy and 35–40 Gy groups in 2/9 (22%) and 5/9 (56%) cases at day 4 and in 4/9 (44%) and 8/9 (89%) cases at day 28, respectively. $BWT_{ratio}^{{V_{ref} }}$ increased on average by 1.32 ± 0.41 and 1.30 ± 0.21 times after 25–30 Gy and of 1.47 ± 0.29 and 1.90 ± 0.83 times after 35–40 Gy at days 4 and 28 respectively. The variations in each group were found to differ significantly from those in the others (*p* < 0.022).

**Table 1:** Results: the descriptive statistics refer to the mean bladder wall thickness normalized to the baseline value and is expressed in arbitrary units

Group	Parameters	Day 4	Day 28
Control (n = 6 rats)	Mean ± SD	0.94 ± 0.13	0.94 ± 0.10
	Median (Min–Max)	0.9 (0.78–1.11)	0.93 (0.81–1.10)
25–30 Gy (n = 9 rats)	Mean ± SD	1.32 ± 0.41	1.30 ± 0.21
	Median (Min–Max)	1.22 (0.82–2.27)	1.29 (1.00–1.73)
	Events above effect size	2/9 (22%)	4/9 (44%)
	*p* value (vs. control group)	< 0.0001	
35–40 Gy (n = 9 rats)	Mean ± SD	1.47 ± 0.29	1.90 ± 0.83
	Median (Min–Max)	1.45 (1.15–1.94)	1.69 (1.02–3.60)
	Events above effect size	5/9 (56%)	8/9 (89%)
	*p* value (vs. control group)	< 0.0001	
	*p* value (vs. 25–30 Gy group)	0.022	

*p* values are reported as result of the Mann–Whitney–Wilcoxon test between groups

## Discussion

To the best of our knowledge, this is the first preclinical study for the in vivo RC evaluation based on a non-invasive US imaging method.

In clinical practice, BWT measurements using ultrasonography are usually performed by measurements on the US image of one or more selected bladder sites of interest according to the physician’s experience [16, 22]. Some authors have proposed using the profile plot of the bladder wall or even the 3D bladder reconstruction [18] to obtain an exact measurement of the space between the two hyper-echoic layers, representing the urothelium and the perivesical tissue [16]. However, all these techniques have two aspects in common: firstly, the hypothesis that the bladder disease can be evaluated by means of a few local measurements and, secondly, strong operator dependency.

**Fig. 5:**
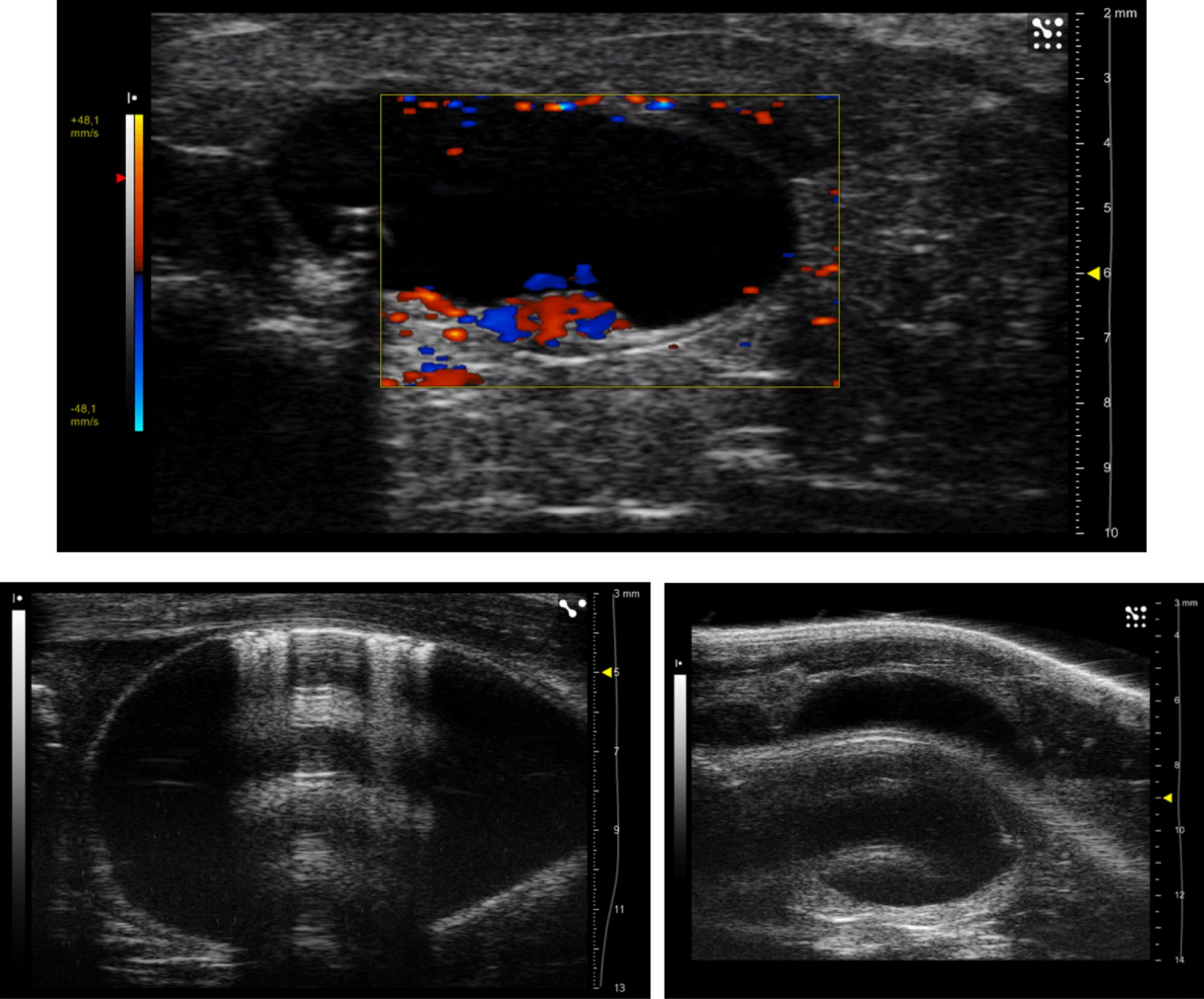
**a** Ultrasound image of a transverse section of a rat bladder using the Doppler mode: red and blue colors evidence the presence of blood vessels which thicken the bladder wall. **b** Artifacts in the ultrasound image due to air bubbles accidently injected by catheter (on the left) and due to tissue in the superficial layers characterized by high acoustic impedance (on the right)

The procedure described in the current work is based on the measurements of an area (instead of segments) in sagittal scans; it was developed to minimize the dependence on delineation. Our approach reduces the impact of anatomical features (see below) possibly leading to a local thickening of the bladder wall, and to misleading interpretations, especially if few transverse scans are considered. As an example, Fig. 5a shows a US image in Doppler mode that reveals the presence of blood vessels thickening the bladder wall in this specific transverse section.

Very interestingly, the trend plotted in Fig. 3 reproduces, in rats, a previously reported clinical finding [17, 19]: in fact, Oelke and Wijkstra have shown that BWT in humans decreases rapidly up to the filling of bladder at about 250 mL, thereafter remaining almost constant until the maximum bladder capacity. We observed the same pattern for rats when the bladder was filled above 1.5 mL, suggesting that the optimal bladder filling for BWT measurements is in the constant region over 1.5 mL, with a confidence of about 15%. The rat bladders studied in the second phase of the experiment were always filled over this limit, with the exception of two cases in the higher dose group in which the radiation damage probably impaired the bladder reservoir capacity. A hyperbaric-like curve trend is confirmed by measurements on both the ventral and dorsal sides. Dorsal BWT values were nearly always lower, likely due to the gravity effect of the overlying bladder, as the measurement was performed in the supine position.

It could be stated that the ultrasound measurements on the dorsal side were probably more biased than those on the ventral side. For example, Fig. 5b shows two typical artifacts: anechogenic acoustic cone due to air bubbles accidently injected from the catheter, and tissue echo with high acoustic impedance in the superficial layers. In both cases the BWT measurement was only compromised on the dorsal side; thus, the ventral side should be preferred for BWT evaluations.

Another point outlined in Fig. 3 is the proportion $BWT_{{mean}} \div 1/\sqrt[3]{V}$, which can be rewritten in the approximation of a spherical organ as $BWT_{mean} \div 1/R$, where $V$ and $R$ are bladder volume and radius, respectively. This dependence is in agreement with the correction proposed by Ke and Kuo [19] to convert the BWT value to that of a reference volume. The application of the correction for bladder filling and the normalization of the measurement to the baseline value are useful adjustments in order to minimize the biological variability component as much as possible in the data analysis.

**Fig. 6:**
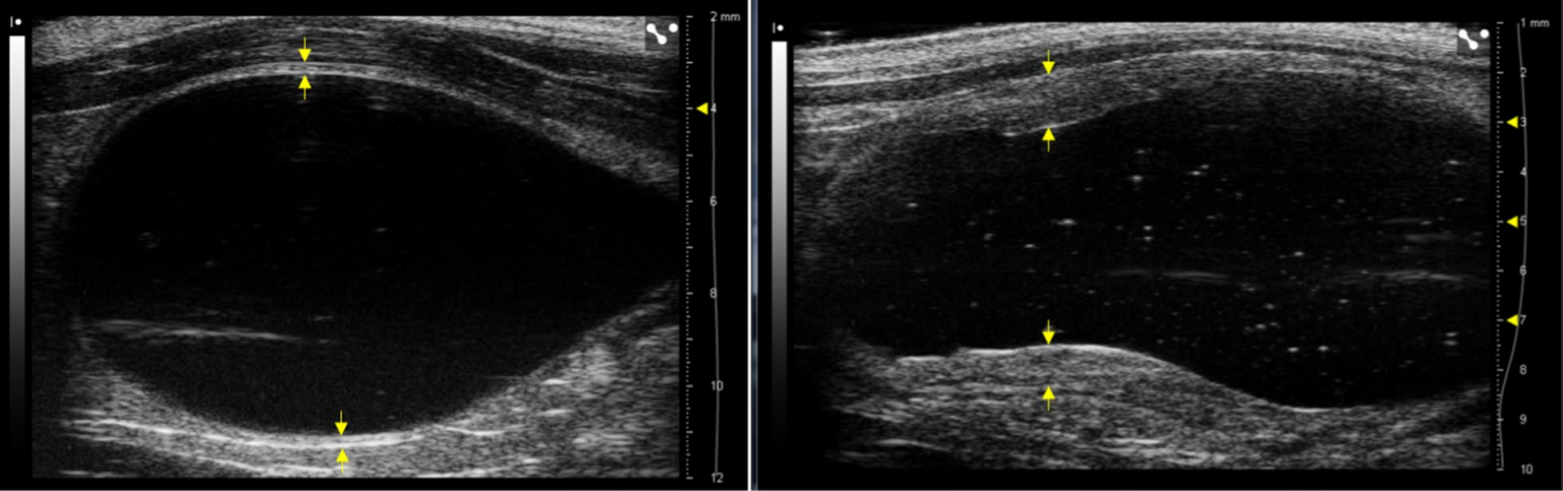
Bladder wall thickness before 1 day (on the left) and after 4 days (on the right) a single radiation dose of 40 Gy

With respect to the second part of the experiment, the major finding was the evidence of a dose–response effect for radiation-induced BWT. As an example, Fig. 6 shows the sagittal scan of the same rat bladder before and after a single radiation dose of 40 Gy: as can be seen, the effect is macroscopic. The plot in Fig. 6 clearly suggests that bladder wall thickening was related to the dose in terms of frequency and intensity of irradiation (i.e. it was time and dose-dependent). Radiation doses ranging between 25 and 30 Gy may be considered to be more representative of clinical doses in a conventional radiotherapy setting in humans [23], while the 35 and 40 Gy range should depict a more extreme situation aiming to better investigate the effect. It is important to underline here that considering the possible differences in terms of radiosensitivity between human and rat bladders, it would not be sensible to use the same range of clinical doses. As further discussed below, the intention of the current study was mainly to elucidate the mechanics of actions of RC. Dose escalation here correlated to an increased frequency and intensity of BWT changes. The sample irradiated with 35–40 Gy showed a faster kinetic of radiation injury, as demonstrated by the 56% of events at day 4 and the much larger $BWT_{ratio}^{{V_{ref} }}$, increased by 1.47 ± 0.29, after 4 days. The groups treated at different doses exhibited significant differences in terms of $BWT_{ratio}^{{V_{ref} }}$ relative to both the control group (*p* < 0.0001) and each other (*p* = 0.022), as reported in Table 1.

Rajaganapathy et al*.* [10] also compared the effect caused by different doses in a rat model, and found that 40 Gy induces stronger changes than 20–30 Gy in terms of reduction of inter-micturition interval as assessed using metabolic cages, as well as degenerative epithelial damage as measured by histochemical analyses. Basically, 35–40 Gy represents a radiation dose that is highly efficient in inducing radiation injuries in rat bladder, although it is likely to be well above the corresponding doses delivered in the human clinical setting. The use of such high doses in specific animal models could nevertheless be useful for a better understanding of the underlying biological mechanisms.

Further studies are needed to confirm these findings, which were based on a small number of animals. Firstly, the correlation of the results with immunohistochemical analyses is mandatory: if such a correlation exists, US imaging will prove to be a powerful tool for the quantification of RC. Furthermore, a parallel study with functional cystometric assessment is necessary to complete the validation of the method and to confirm the observational timing of the acute damage, which was chosen relying on already published data [2, 3]. Lastly, it should be noted that the non-invasiveness of the described method could be slightly altered by catheterization, and could be severely undermined when male rats are used. In fact, while female rats can be subjected to transurethral catheterization, males must undergo a surgical intervention to insert the catheter into the bladder (the catheter is tunneled subcutaneously to the inter-scapular area) due to a different anatomical conformation of the urethra. This is, however, a well-known problem common to other research tools, such as the widely used cystometry.

## Conclusion

A preclinical rat model of radiation cystitis induced by a small animal irradiator platform was assessed in the acute phase using US imaging. Bladder filling above 1.5 mL was found to be the optimal condition to measure bladder wall thickness, since BWT does not vary with further filling. In addition, the ventral layer should be preferred when assessing BWT as it is less prone to measurement biases. The delivery of 35–40 Gy in a single fraction induced more evident BWT changes than 25–30 Gy, with a significantly increased BWT. Our model seems to provide a fast and reliable way to measure and compare BWT, taking into account inter- and intra-case variability. Although preliminary, these results are promising, addressing the potential of this non-invasive approach in quantifying radiation-induced effects on the bladder in rat models.

The preliminary findings of the current work will be validated by immunohistochemical analysis in the coming months, aiming to correlate BWT changes with late fibrotic effects. The euthanizing time suggested by the literature to observe morphological and histological alterations in the chronically damaged bladder tissue is at least 6 months [12, 15]. Future investigations will focus on the correlation with urinary functionality evaluated by means of transurethral cystometry and also on the possible effect of isoflurane on rat bladder function by using different anesthesia agents like urethane [24]. Ultrasound could also be employed to assess the changes in vascularization using the contrast enhanced ultrasound (CEUS) technique [25–27], but ad hoc development of the animal model will be necessary. Finally a larger and blinded study including non-treated and radiated rats will be performed in order to demonstrate the reliability of the ultrasound BWT measurement method described in this work.

## Data Availability

The datasets analysed during the current study are available from the corresponding author upon reasonable request.

## Abbreviations

RC: Radiation cystitis
RT: Radiotherapy
US: Ultrasound
BWT: Bladder wall thickness
IACUC: Institutional Animal Care and Use Committee
*V*: Ellipsoid volume
abc: Ellipsoid axes
(*BWT*_*mean*_): Mean bladder wall thickness
*BWA*: Bladder wall area across segment of 4 mm
2*R*_*mean*_: Mean diameter
($BWT_{ratio}^{{V_{ref} }}$): Mean bladder wall thickness normalized to the baseline value and converted to the corresponding BWT to a hypothetical reference volume
CEUS: Contrast enhanced ultrasound

## References

[1] Zwaans BMM, Chancellor MB, Lamb LE (2016). Modeling and treatment of radiation cystitis. Urology (Internet).

[2] Dörr W, Beck-Bornholdt H-P (1999). Radiation-induced impairment of urinary bladder function in mice: fine structure of the acute response and consequences on late effects. Radiat Res (Internet).

[3] Jaal J, Brüchner K, Hoinkis C, Dörr W (2004). Radiation-induced variations in urothelial expression of intercellular adhesion molecule 1 (IGAM-1): association with changes in urinary bladder function. Int J Radiat Biol (Internet).

[4] Jaal J, Dörr W (2006). Radiation induced inflammatory changes in the mouse bladder: the role of cyclooxygenase-2. J Urol (Internet).

[5] Basford JR (2002). The Law of Laplace and its relevance to contemporary medicine and rehabilitation. Arch Phys Med Rehabil.

[6] Roccabianca S, Bush TR (2016). Understanding the mechanics of the bladder through experiments and theoretical models: where we started and where we are heading. Technology.

[7] Chai X, Van Herk M, Van De Kamer JB, Hulshof MCCM, Remeijer P, Lotz HT (2011). Finite element based bladder modeling for image-guided radiotherapy of bladder cancer. Med Phys.

[8] Chai X, Van Herk M, Hulshof MCCM, Bel A (2012). A voxel-based finite element model for the prediction of bladder deformation. Med Phys.

[9] Lundbeck F, Ulsø N, Overgaard J (1989). Cystometric evaluation of early and late irradiation damage to the mouse urinary bladder. Radiother Oncol (Internet).

[10] Rajaganapathy BR, Janicki JJ, Levanovich P, Tyagi P, Hafron J, Chancellor MB (2015). Intravesical liposomal Tacrolimus protects against radiation cystitis induced by 3-beam targeted bladder radiation. J Urol (Internet).

[11] Zwaans BMM, Krueger S, Bartolone SN, Chancellor MB, Marples B, Lamb LE (2016). Modeling of chronic radiation-induced cystitis in mice. Adv Radiat Oncol (Internet).

[12] Vale JA, Bowsher WG, Liu K, Tomlinson A, Whitfield HN, Trott KR (1993). Post-irradiation bladder dysfunction: development of a rat model. Urol Res (Internet).

[13] Stewart FA (1986). Mechanism of bladder damage and repair after treatment with radiation and cytostatic drugs. Br J Cancer (Internet).

[14] Jaal J, Dörr W (2005). Early and long-term effects of radiation on intercellular adhesion molecule 1 (ICAM-1) expression in mouse urinary bladder endothelium. Int J Radiat Biol (Internet).

[15] Crowe R, Vale J, Trott KR, Soediono P, Robson T, Burnstock G (1996). Radiation-induced changes in neuropeptides in the rat urinary bladder. J Urol (Internet).

[16] Blatt AH, Titus J, Chan L (2008). Ultrasound measurement of bladder wall thickness in the assessment of voiding dysfunction. J Urol.

[17] Oelke M, Wijkstra H (2006). Ultrasound detrusor wall thickness measurements to diagnose Bladder Outlet Obstruction in men. Urodinamica.

[18] Chalana V, Dudycha S, Yuk J-T, McMorrow G (2005). Automatic measurement of ultrasound-estimated bladder weight (UEBW) from three-dimensional ultrasound. Rev Urol.

[19] Ke QS, Kuo HC (2011). The promise of bladder wall thickness as a useful biomarker for objective diagnosis of lower urinary tract dysfunction. Tzu Chi Med J (Internet).

[20] Schneider CA, Rasband WS, Eliceiri KW (2012). NIH Image to ImageJ: 25 years of image analysis. Nat Methods.

[21] Van Hoof SJ, Granton PV, Verhaegen F (2013). Development and validation of a treatment planning system for small animal radiotherapy: SmART-Plan. Radiother Oncol (Internet).

[22] Bala KG, Chou YH (2010). Ultrasonography of the urinary bladder. J Med Ultrasound (Internet).

[23] Clement CH, Stewart FA, Akleyev AV, Hauer-Jensen M, Hendry JH, Kleiman NJ (2012). ICRP publication 118: ICRP statement on tissue reactions and early and late effects of radiation in normal tissues and organs—threshold doses for tissue reactions in a radiation protection context. Ann ICRP.

[24] Smith PP, Deangelis AM, Kuchel GA (2012). Evidence of central modulation of bladder compliance during filling phase. Neurourol Urodyn.

[25] Chan ESY, Patel AR, Larchian WA, Heston WD (2011). In vivo targeted contrast enhanced micro-ultrasound to measure intratumor perfusion and vascular endothelial growth factor receptor 2 expression in a mouse orthotopic bladder cancer model. J Urol (Internet).

[26] Nicolau C, Bunesch L, Sebastia C, Salvador R (2010). Diagnosis of bladder cancer: contrast-enhanced ultrasound. Abdom Imaging.

[27] Ge W, Zheng Y, Tao Z (2014). Contrast-enhanced ultrasound analysis of tissue perfusion in tumor-bearing mice following treatment with endostatin combined with radiotherapy. Exp Ther Med.

